# In Situ-Reinforced Phase Evolution and Mechanical Properties of CoCrFeNi High-Entropy Alloy Composite Coating on Q235B by Laser Cladding with Nb Addition

**DOI:** 10.3390/ma18071572

**Published:** 2025-03-31

**Authors:** Feimuyun Yang, Zhixuan Xiao, Zehuan Chen, Hongtao Jin, Chao Gao, Jiang Huang

**Affiliations:** 1 College of Electronics and Information Engineering, Guangdong Ocean University, Zhanjiang 524088, China; 11911324ee1@stu.gdou.edu.cn (F.Y.); xzx2331231234@163.com (Z.X.); 13622427639@163.com (Z.C.); j779730543@163.com (H.J.); 2 Guangdong Provincial Key Laboratory of Intelligent Equipment for South China Sea Marine Ranching, Guangdong Ocean University, Zhanjiang 524088, China

**Keywords:** laser cladding (LC), in situ reaction, microstructure, hardness, wear, corrosion, HEA

## Abstract

Q235B is widely used in marine engineering materials; however, its wear resistance and corrosion resistance are poor. To improve wear and corrosion resistance, a CoCrFeNi high-entropy alloy (HEA) composite coating was cladded using laser cladding (LC) technology. Different proportions of tungsten carbide (WC) and Nb elements were added to the CoCrFeNi HEA coating, and the microstructure, phase, hardness, wear, and corrosion resistance of three different composite coatings were analyzed. The results show that the in situ synthetic phase is composed of Face central cubic (FCC) (Cr_3_C_2_) and strengthening phases such as W, WC, and NbC. In the process of LC, Nb will react with WC in situ, which not only reduces the morphology of the CoCrFeNi HEA cladding coating changed by adding WC, but also generates NbC, which leads to the dissolution of WC particles and improves the uniformity of particle distribution of the coating. The hardness of the coating with Nb is increased by 1.40 times, the wear resistance is enhanced, and the peeling of the hard phase is reduced during wear. The corrosion resistance of the coating with only WC particles is the best. Nb reduces the morphology of CoCrFeNi HEA cladding coating changed by WC particles. Although the coating with Nb is not as strong as that with WC particles only, it has outstanding hardness and wear resistance.

## 1. Introduction

Q235B is widely used in welded structural parts with high quality requirements in building engineering because of its good toughness, castability, and low price, but its bad wear and corrosion resistance shorten the service life of welded components and bring risks to engineering buildings [1,2,3,4]. In order to improve the wear resistance and corrosion resistance of Q235B and reduce the loss caused by wear and corrosion, a wear- and corrosion-resistant coating needs to be prepared on its surface. Common surface modification technologies include magnetic sputtering [5,6], thermal spraying [7], Melt insert-gas welding; Tungsten insert-gas welding (MIG/TIG) [8], vacuum sintering [9], and LC [10], Among these methods, LC is widely used because of its environmental friendliness as it does not cause pollution, easy processing, and high efficiency [11,12,13,14,15].

HEAs is a new material proposed within the past twenty years [16,17,18]. Their unique structure makes HEAs have high corrosion resistance and excellent mechanical properties [19]. CoCrFeNi HEAs have been widely studied because of their face-centered cubic (FCC) structure and excellent ductility and fracture. However, the mechanical characteristics of CoCrFeNi HEAs are not outstanding [20].

It is common practice to add hard WC particles to improve HEAs’ properties [21,22,23,24,25]. Huang Y et al. [26] found that decomposed WC can strengthen the cladding coating of CoCrFeNi with solid solution so as to enhance the hardness, wear, and corrosion resistance of CoCrFeNi HEAs.

The CoCrFeNi/WC HEA cladding coating prepared by LC can only slightly dissolve WC particles, and the distribution of WC particles is uneven [27,28,29]. Hong S et al. [30] discovered that the strong carbide element Nb can promote the dissolution of dissolved WC. Wu H et al. [31] found that NbC can improve the wear resistance of the coating. Therefore, it is a scientific supplement to previous research whether the strong carbide element Nb, which can promote in situ reaction, can be added to the CoCrFeNi HEA/WC cladding coating to adjust the content of elements and further control the structure of an in situ-reinforced phase to generate NbC so as to promote the full dissolution of WC and improve its inhomogeneity, further enhance the solid solution strengthening effect of WC on the CoCrFeNi cladding coating, and improve its properties.

In this paper, LC technology was used to make the CoCrFeNi HEA cladding composite coating by adding commonly used hard-phase WC particles and the strong carbide element Nb to promote the in situ reaction in turn. The mechanical properties of the composite coating were studied.

## 2. Experiment

### 2.1. Materials

Q235B with dimensions of 100 mm (length) × 50 mm (width) × 2 mm (thickness) was used as the base material, which was supplied by Sichuan Quanyue metal material co., ltd, Sichuan, China. The main chemical components of Q235B mild steel are shown in Table 1.

**Figure 1:**
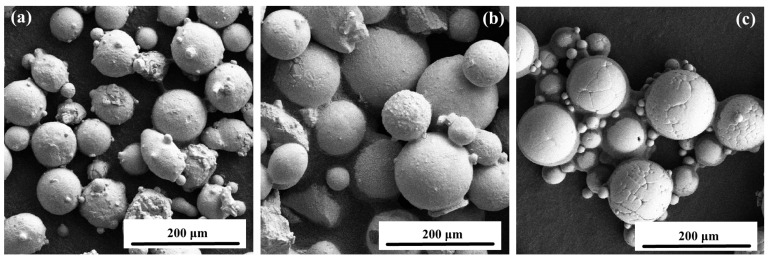
SEM images of (**a**) CoCrFeNi HEA, (**b**) WC, and (**c**) Nb particle powders.

Before LC, Q235B was polished, and the stain was cleaned with alcohol. The LC material is a mixture of CoCrFeNi HEA, WC, and Nb powders in specific proportions, which were provided by Sichuan Quanyue metal material co., ltd, Sichuan, China. SEM images of CoCrFeNi HEA powder, spherical WC particle, and Nb powder are shown in Figure 1; the CoCrFeNi HEA powder is polygonal spherical, WC powder is spherical or nearly spherical, and Nb powder is spherical with small particles.

In order to study the effects of WC and Nb on the microstructure and properties of the coatings, specific contents of WC and Nb were added in sequence during the powder configuration process. The compositions of three coatings in the experiment are shown in Table 2. Before LC, the powders were prepared in groups and then evenly mixed using a mechanical powder mixer (model: F-P400; manufacturer: Beijing Grinder Instrument Co., Ltd., Beijing, China), and the mixed powder layer with a thickness of 2.0 mm was spread on the substrate.

### 2.2. LC Technology

For this experiment, we used the XL-F2000W fiber continuous laser processing system (model: XL-F2000W, manufacturer: Maxphotonics Co. Ltd., Shenzhen, China); the laser output power was 14 kw, a circular Gaussian beam was used, and the spot diameter was 2.5 mm. A multi-track coating was prepared. The wavelength of the laser beam was 1080 nm, the preset powder mode was adopted in the experiment, the laser scanning speed was 500 mm/min, the overlapping rate was 40%, the defocusing amount was +5 mm, and the powder coating thickness was 2 mm using a standard mold. Before LC, pure Ar protection gas with 99.99% purity was injected at a rate of 5 L/min.

### 2.3. Text Procedure

A wire cutter (model: DK77; manufacturer: Taizhou Ruite mechanical equipment Co., Ltd.) was used to cut the cladding coating to 10 mm × 10 mm × 2 mm, 10 mm × 5 mm × 2 mm and 15 mm × 15 mm × 2 mm. Firstly, we used 60-mesh sandpaper to roughly polish the sample and then polished the sample’s surface with 400 mesh, 600 mesh, 800 mesh, 1200 mesh, 1500 mesh, and 2000 mesh until the sample’s surface was smooth. The sandpaper used is made of SiC. The longitudinal profile mechanical properties and corrosion properties were analyzed.

Using scanning electron microscopy (SEM, FE1, Quanta250FEG, Hillsboro, OR, USA) and an optical microscope (OM, XIL-302/302BD, Yuelian optical instrument Co., Ltd., Guangzhou, China), we analyzed the longitudinal profile microstructure. The chemical composition of the samples was determined using an energy-dispersive spectrometer (EDS, Quanta250FEG, Hillsboro, OR, USA). Using an X-ray diffractometer (XRD, XRD-6100, Shimadzu, Kyoto, Japan), the scanning speed was 2°/min, and the composite was studied as well as the phase composition characteristics of the LC coating. We selected a scanning angle ranging from 20° to 80° according to the actual situation.

Coating cross section hardness test was conducted using Vickers hardness tester (model: MHVD-1000AT, manufacturer: Yizong Precision Instrument Co., LTD. Shanghai, China). Experimental parameters were as follows: load of 200 g and load duration of 10 s. Point spacing was 0.2 mm, and each sample was measured in 3 columns and averaged. The wear resistance of the coated surface was tested using a pin-disk friction and wear testing machine with a displacement sensor (model: SFT-2M; manufacturer: Lanzhou Zhongkehua Technology Development Co., LTD., Lanzhou, China). We used a 6 mm GCr15 steel ball as a counterpart for wear conditions. The test parameters were set to a load of 20 N, a rotating radius of 2 mm, a rotating speed of 200 r/min, and a test duration of 1 h.

In the electrochemical workstation (CHI660E, Chenhua Instrument, Shanghai, China), the conventional three-electrode method was used to test the corrosion property of the sample soaked in 3.5 wt% NaCl for 1 h at room temperature. Electrochemical impedance spectroscopy (EIS) was used to obtain the impedance spectrum data at the frequency of 10–1~10–5 HZ. Tafel data were obtained at a test voltage ranging from −0.5 V to + 0.375 V.

## 3. Results

### 3.1. Macroscopic and Microscopic Observations

**Figure 2:**
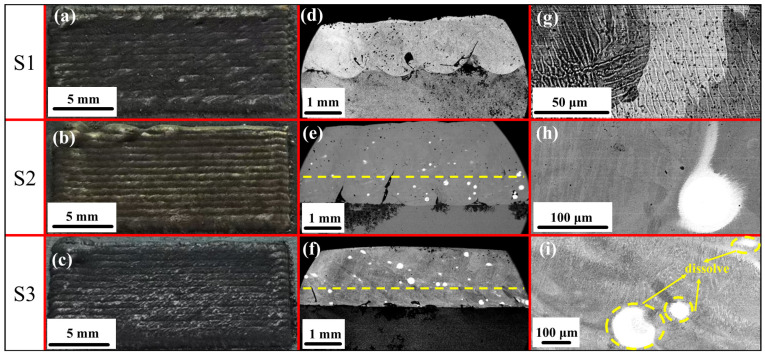
Macroscopic and microscopic observations of coating: (**a**–**c**) surface of CoCrFeNi HEA composite coating; (**d**–**f**) microscopic cross sections of CoCrFeNi HEA composite coating; (**g**–**i**) composite coating segment.

The LC CoCrFeNi HEA composite coating is shown in Figure 2a–c. It can be seen that the LC coating has a smooth surface and no obvious pores or hot cracks, and the cladding coating is completely cladded on the substrate.

**Figure 3:**
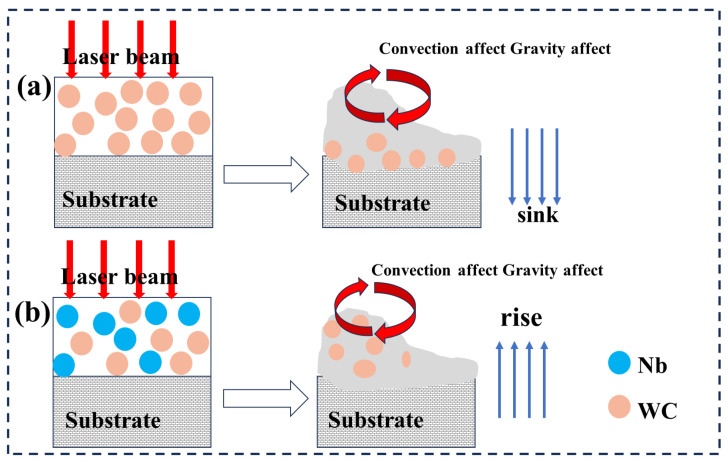
Convection and gravity models during coating solidification. (**a**) In coating S2, WC particles sink to the bottom under the influence of convection and gravity. (**b**) In coating S3, the influence of convection and gravity is small, and WC particles are evenly distributed in the coating.

The microscopic cross section of the composite coating is shown in Figure 2d,e. It can be seen that large WC particles are mostly distributed in the lower part of S2 of the composite coating, while small particles are not evenly distributed. Figure 2f shows that WC particles mostly gather in the middle and upper parts of composite coating S3, and the number of particles decreases compared with Figure 2e. This phenomenon can be attributed to convection and gravity, as shown in Figure 3. Due to the large weight of WC particles, they are greatly affected by gravity and gas convection and sink to the bottom, as shown in Figure 3a. As shown in Figure 3b, an in situ reaction occurred between Nb and WC, and the large particles of WC decomposed partially, while the small particles decomposed completely, resulting in a decrease in the weight of WC particles and a small impact of gravity and gas convection, resulting in WC particles not moving downward before the solidification of the cladding coating. Therefore, after the addition of Nb, the large WC particles in the microscopic cross section are mainly divided in the upper part, and the WC particles are reduced [32,33,34].

**Figure 4:**
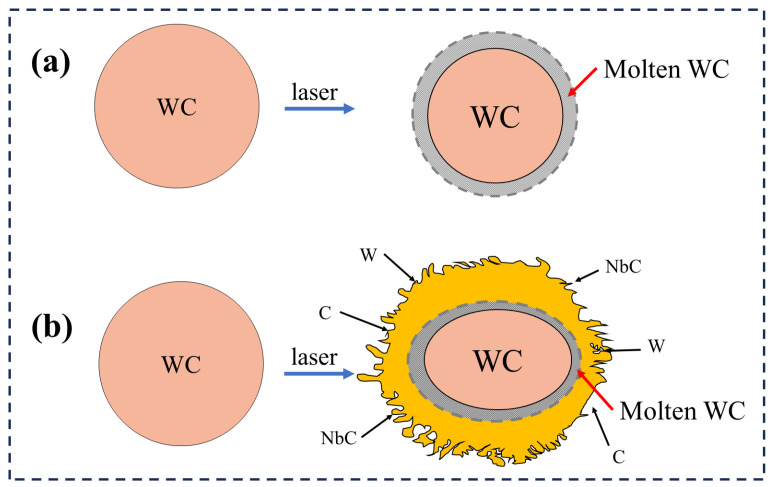
WC decomposition diagram. (**a**) In coating S2, WC particles are partially decomposed; (**b**) in coating S3, WC particles are severely decomposed.

Figure 2g–i show the microstructures of the composite coatings. It can be seen that the S1 coating is dendritic, and S2 contains WC particles, which are mostly spherical and nearly spherical, while WC particles are oval and flat after adding Nb. Figure 2h shows that WC particles are partially decomposed, while small particles of WC are seriously decomposed and large particles are partially decomposed after adding Nb. To further show the decomposition of WC particles, Figure 4 illustrates a schematic diagram of WC decomposition. Figure 4a shows the partial decomposition of WC particles. It can be observed that after laser heating, the WC particles exhibit only slight decomposition while maintaining a spherical shape overall. Figure 4b depicts severe decomposition after the addition of Nb, where WC particles appear elliptical with irregular edges. By comparing Figure 4a,b, it can be inferred that an in situ reaction occurred between Nb and WC, forming NbC and W, which significantly promote the decomposition of WC particles.

**Figure 5:**
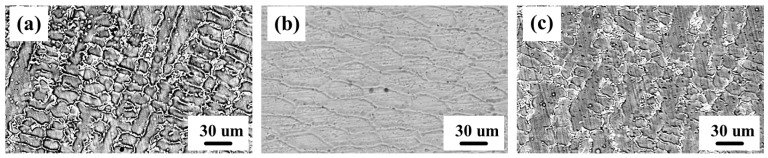
SEM diagrams of coating: (**a**) S1; (**b**) S2; and (**c**) S3.

Figure 5a,b are the microstructures of the composite coatings. The microstructure of S1 is dendritic, mostly containing short columnar crystals and a few long columnar crystals. The grain growth direction is isotropic. After adding WC, the microstructure changes into cellular crystal, and the grain growth direction is isotropic and uniform, as shown in Figure 5b. With the addition of Nb, the microstructure changes back to a dendritic shape. However, compared with the microstructure of the S1 coating, the proportion of long columnar crystals increased. The results of SEM show that the addition of Nb decomposes WC and changes the microstructure of WC back to dendritic.

### 3.2. XRD Analysis

**Figure 6:**
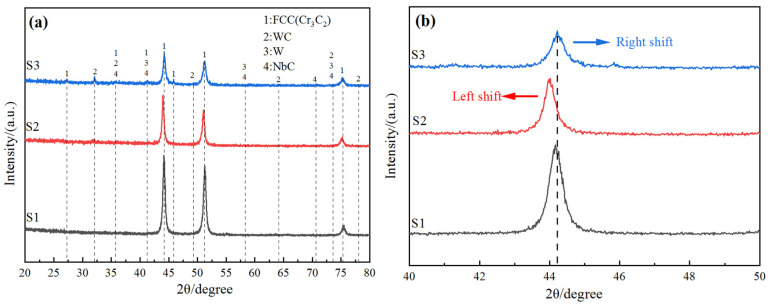
XRD pattern of coating. (**a**) Overall; (**b**) main peak 1.

The results of the XRD analysis are shown in Figure 6. One can see that the in situ synthetic phase in the molten pool is mainly composed of FCC (Cr3C2) (JCPDS: 35-0804) and strengthening phases such as W (JCPDS: no. 04-0806), WC (JCPDS: no. 25-1047), and NbC (JCPDS: no. 38-1364). There is NbC in coating S3, which further proves that NbC is generated after an in situ reaction between Nb and WC. This is similar to the research results of Hong S et al. [30]. The main component of the composite coating is HEA. After adding WC particles, there is a small amount of W precipitated phase in the cladding coating. Diffraction peaks of 35.88°, 41.22°, 58.2°, 70.66°, and 73.54°, which were not observed in the control coating (S1) with a diffraction angle of 2θ, were generated. In addition, it is observed that WC and NbC appear at the same peak position because under the same proportions of WC and Nb powder added in S3, Nb has strong reducibility, and a small amount of Nb reacts with C in CoCrFeNi HEA in situ, leaving a small amount of WC, which leads to the phenomenon where WC and Nb are at the same peak position.

Figure 6b shows the XRD spectra of the main peaks. It is shown that the diffraction peak of WC particles is slightly shifted to the left relative to the main peak of coating S1. According to Bragg’s law, the left shift in the peak indicates the increase in lattice spacing, which is caused by lattice distortion [35,36]. After Nb was added, the diffraction peak shifted slightly to the right relative to the peak of coating S2, which was almost consistent with the peak position of coating S1. The reason for this is that the in situ reaction occurred between Nb and WC, so most of WC melted, which led to the peak value moving back.

**Figure 7:**
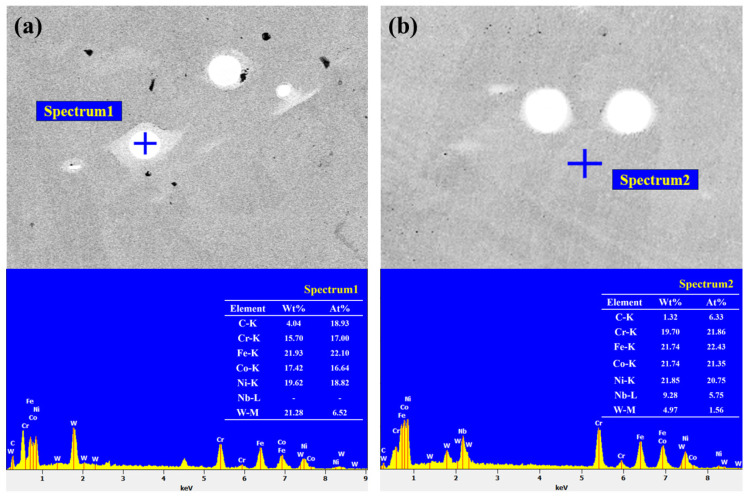
The element composition of the precipitated phase in the coatings: (**a**) S2; (**b**) S3.

Figure 7 shows the energy spectrum of the precipitated phase of the coatings. It was found that coatings S2 and S3 had similar compositions. As a strong carbide-forming element, Nb has a much higher binding force with C than W [30]. In the process of high temperature melting and cooling, Nb and C combine with each other to form NbC. With the addition of Nb, the contents of C and W decreased obviously, and a new precipitated phase NbC was formed through decomposition and synthesis.

**Figure 8:**
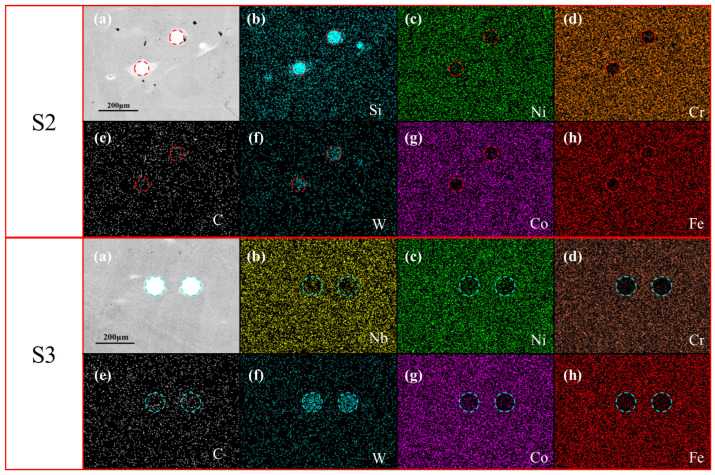
Element distribution of primary precipitated phase in S3 coating. (**a**) SEM images of S2 and S3, and the corresponding EDS mapping, (**b**) Si, (**c**) Ni, (**d**) Cr, (**e**) C, (**f**) W, (**g**) Co, (**h**) Fe.

Figure 8 shows EDS images of the S2 and S3 coatings. It is seen that the precipitated phases in the S2 and S3 coatings are spherical. The precipitated phase of coating S2 contains almost no Cr, Co, and Fe, and small amounts of Ni and W are enriched. In coating S3, W is abundant, and Nb and Cr are found in small concentrations. This shows that the strong carbon compound Nb competes with weak carbides such as W and Co, which intensifies the precipitation of W and Cr.

In order to verify that the strong carbon compound Nb has stronger binding ability with C than Cr and W, NbC is generated. Thermodynamics is used to predict the possibility of chemical reaction in a molten pool [37,38].

3Cr + 2C→Cr_3_C_2_ (1)

W + C→WC(2)

Nb + C→NbC(3)

**Figure 9:**
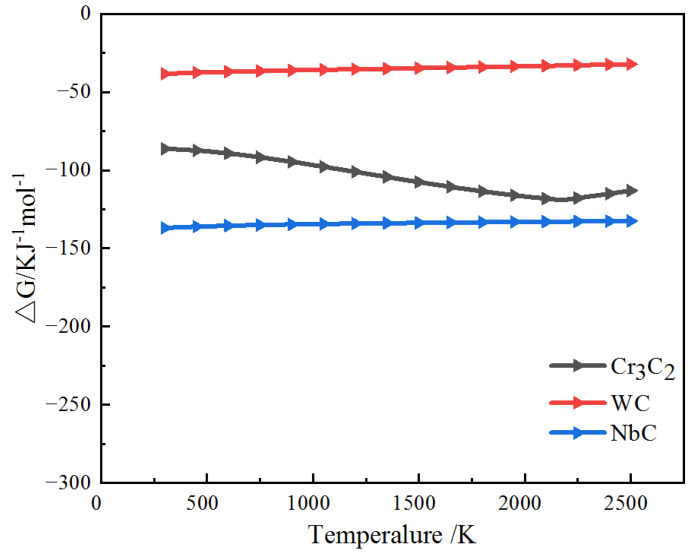
Change in standard Gibbs free energy.

It is known that when the Gibbs free energy (△G) is less than 0, a reaction can occur at this temperature. The smaller the Gibbs free energy (△G), the more likely it is to occur [39]. Figure 9 shows the AG data of these images at 300–2500 K. The AG values of these reactions are all less than 0, that is, these reactions can all occur spontaneously. At 300–2500 K, the △G of NbC is the lowest, indicating that NbC has the largest tendency to form in the melt pool, further indicating that Nb more easily combines with C to form NbC than Cr and W.

### 3.3. Hardness and Wear Properties

**Figure 10:**
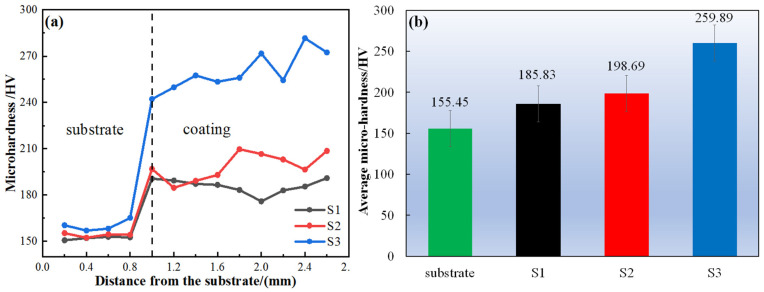
(**a**,**b**) show the hardness and average hardness of the substrate and coatings.

Figure 10 expresses the results of hardness and average hardness of the composite coating. It can be seen that the hardness of the substrate has no obvious change, which is about 155.45 HV, while the hardness is obviously improved at the interface between the substrate and the coating. The average hardness of the coating increases gradually with the addition of WC and Nb particles, respectively. The hardness of S3 is the highest, being 1.67 times higher than that of the substrate. The sample with Nb has the highest hardness. The addition of Nb promoted the decomposition of WC particles in the CoCrFeNi HEA/WC cladding layer, which made the composite coating elements more uniform, thus improving the hardness of the composite coating [30].

**Figure 11:**
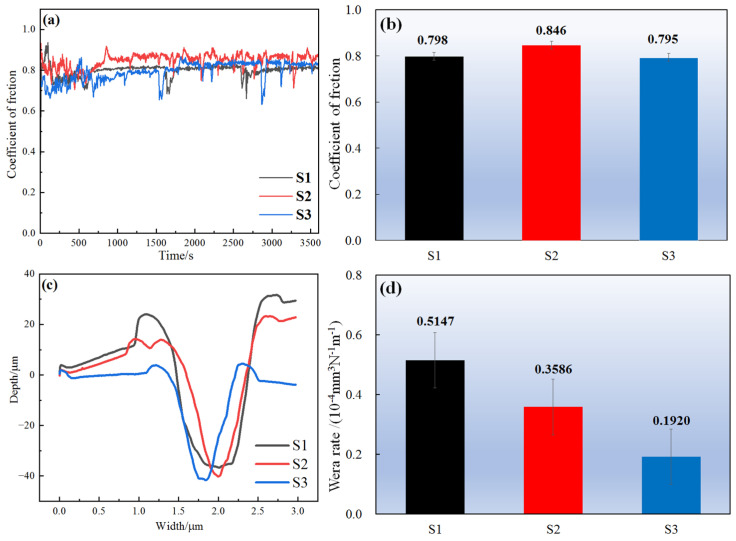
(**a**,**b**) show the COF and average COF of the coating; (**c**) shows the wear profile of the coating; (**d**) shows the wear rate of the coating.

The friction and wear characteristics are shown in Figure 11a–d. Figure 11a,b show the coefficient of friction (COF) of the sample and its average COF, among which S3 has the lowest COF. When the wear distance is 2984 mm, coating S3 has the lowest COF at only 0.705. The wear profile of each layer is shown in Figure 11c, in which the wear profile of coating S3 is the shallowest and narrowest, indicating that S3 has good wear performance. Figure 11d shows the wear rate of the coating. It was found that the wear rate of the coating gradually decreases with the addition of WC and Nb, and S3 has the lowest wear rate, showing the best wear resistance. Therefore, it is seen that the adding Nb to the CoCrFeNi HEA/WC cladding coating, produced in the process of LC, enhances the wear resistance of the cladding coating [31].

**Figure 12:**
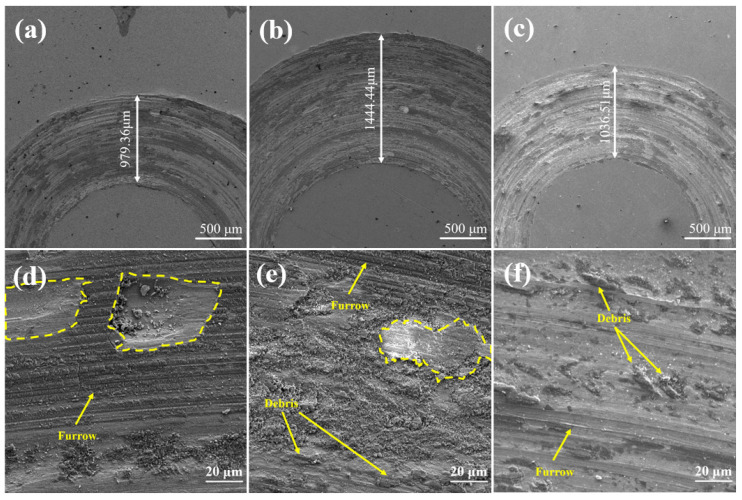
(**a**–**c**) SEM images of the wear profile of the coating: (**a**) S1; (**b**) S2; and (**c**) S3. (**d**–**f**) SEM images of friction between the coating wear surface of the coating and GCr15 steel: (**d**) S1; (**e**) S2; and (**f**) S3.

The friction and wear profile of the composite coating are shown in Figure 12a–c. Coating S2 has the largest transverse grain diameter of 1.444 mm, and coating S1 has the smallest transverse grain diameter of 0.979 mm. However, the cross-grain diameter of S3 is close to that of S1, which indicates that an in situ reaction occurs in coating S3, and the cross grain is closer to S1 when it is worn.

During the rubbing process of coating S1 (Figure 12d) and coating S2 (Figure 12e), the abrasive delamination dust falls off, leaving peeling pits with different depths. When sliding on the friction pair, deep scratches were left on the two coatings. Coating S1 has the most and complete peeling pits and deep scratches. The scratches on coating S2 are the deepest, and in the process of friction, a large area of debris and a complete peeling pit appear, indicating that the COF increases after coating S2 is worn off by one coating. However, in Figure 12f, the scratch of coating S3 is the shallowest, and no obvious peeling pit is found. Combined with the data of the wear test, coating S3 has the best wear performance.

### 3.4. Corrosion Resistance

The corrosion standard is GB/T 19292 [40].

**Figure 13:**
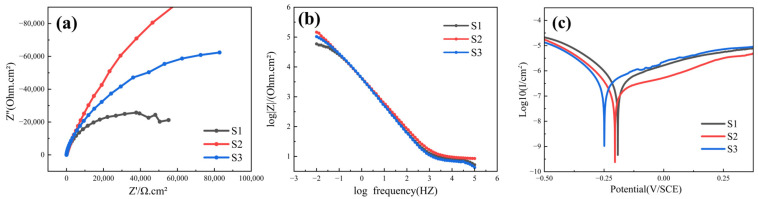
Electrochemistry of coatings: (**a**) Nyquist impedance spectroscopy; (**b**) Bode plot of |Z| and frequency; (**c**) Tafel curve.

Figure 13a shows the Nyquist evolution of coatings of different test groups in 3.5 wt% NaCl solution. In general, the larger the diameter of the Nyquist curve, the better the corrosion resistance of the coating [40,41,42]. It can be seen that the curve diameter from large to small is S2 > S3 > S1, indicating that coating S2 has the best corrosion resistance. Figure 13b shows the bod plot of |Z| and frequency for each sample. Under normal circumstances, the impedance value of the system at a low frequency can well reflect the corrosion resistance of the coating, and the impedance of the system is proportional to the corrosion resistance of the coating. Therefore, the impedance value follows the order of S2 > S3 > S1, which also proves that the corrosion resistance of coating S2 is the best. The corrosion resistance of each sample soaked in 3.5 wt% NaCl solution for 1h was measured by potential polarization, and the results are shown in Figure 13c. It can be seen that all coatings enter a wider passivation zone after spontaneous passivation. In the range of scanning voltage [−0.5, 0.375], no obvious pitting corrosion was observed in any coatings.

The results of self-corrosion potential (Ecorr) and self-corrosion current density (Icorr) of each sample in 3.5%wt NaCl solution are shown in Table 3. It can be seen that the self-corrosion potential (Ecorr) of coating S1 is the smallest, and the self-corrosion current density (Icorr) of coating S2 is the smallest. Since the self-corrosion current density (Icorr) is positively correlated with the corrosion rate of the coating, it can reflect the corrosion resistance of the coating, indicating that the corrosion rate of coating S2 is the lowest and its corrosion resistance is the best, which is consistent with the results of the Nyquist curve and frequency Baud diagram. This is because a small amount of W can improve corrosion resistance, and coating S2 precipitates a small amount of W after LC [43].

## 4. Conclusions

In this experiment, a CoCrFeNi HEA composite coating was prepared on the surface of Q235B by LC. The effects of adding WC and Nb on the microstructure evolution, wear performance, and corrosion resistance of the cladding coating were studied, and the following conclusions were drawn:

(1) The CoCrFeNi HEA/WC cladding coating with added Nb was prepared using LC technology and mainly consisted of FCC, W, WC, and NbC phases. With the addition of WC, the lattice was distorted, and the position of main peak 1 shifted to the left as a whole. With the addition of NbC, the position of the main peak 1 shifted back as a whole. This shows that Nb reacts with WC in situ, which decomposes most of the WC, leading to lattice distortion.

(2) The addition of Nb can enhance the uniformity of the cladding layer. Affected by convection and gravity, undissolved WC particles are mainly distributed at the bottom of the molten pool. However, with the addition of Nb element, the in situ reaction with WC particles promotes the decomposition of undissolved WC particles and makes the particles evenly distributed in the coating.

(3) The addition of Nb has a significant effect on the microstructure of the cladding coating. The microstructure analysis shows that the microstructure changed from dendritic crystal to cellular crystal with the addition of WC. With the addition of Nb, the microstructure changed back to dendritic, but the proportion of long columnar crystals increased.

(4) The addition of Nb can not only improve hardness but also improve wear resistance. It was shown that Nb dissolves most WC particles and generates NbC, which enhances the solid solution strengthening effect of the CoCrFeNi HEA/WC cladding layer. Compared with the CoCrFeNi HEA coating, the hardness of the coating with Nb element is increased by 1.40 times, and the wear rate is reduced by 62.6%, which effectively reduces the peeling of the hard phase during wear.

(5) The improvement in the corrosion resistance of the coating with Nb is not outstanding. The CoCrFeNi HEA/WC cladding coating with the addition of only WC particles has the largest curve, the smallest corrosion current, and the largest impedance, indicating that the CoCrFeNi HEA/WC cladding coating has the best corrosion resistance.

## Figures and Tables

**Table 1:** Chemical compositions of materials (mass fraction%).

Material	Chemical Composition (Mass Fraction)							
	C	Si	Mn	Fe	S	P	Cr	Co
Q235B	0.15	0.15	0.25	Bal	0.11	0.12	-	-
CoCrFeNi HEA	26.16	-	-	24.79	-	-	22.98	26.16

**Table 2:** Composition design of coatings (wt.%).

Number of Coating	CoCrFeNi Powder	WC Powder	Nb Powder
S1	100	0	0
S2	95	5	0
S3	90	5	5

**Table 3:** The samples’ Ecorr and Icorr values.

Materials’	Ecorr (V/SCE)	Icorr (A/cm^2^)
S1	1.91821 × 10^−1^	2.76671 × 10^−9^
S2	2.03847 × 10^−1^	3.62645 × 10^−9^
S3	2.48573 × 10^−1^	2.67767 × 10^−−9^

## Data Availability

The original contributions presented in this study are included in the article. Further inquiries can be directed to the corresponding authors.
